# Enhanced Electrochemical Performance of Sugarcane Bagasse-Derived Activated Carbon via a High-Energy Ball Milling Treatment

**DOI:** 10.3390/nano12203555

**Published:** 2022-10-11

**Authors:** Likkhasit Wannasen, Narong Chanlek, Sumeth Siriroj, Santi Maensiri, Ekaphan Swatsitang, Supree Pinitsoontorn

**Affiliations:** 1 Department of Physics, Faculty of Science, Institute of Nanomaterials Research and Innovation for Energy (IN-RIE), Khon Kaen University, Khon Kaen 40002, Thailand; 2 Synchrotron Light Research Institute (Public Organization), 111 University Avenue, Muang District, Nakhon Ratchasima 30000, Thailand; 3 School of Physics, Institute of Science, Suranaree University of Technology, Nakhon Ratchasima 30000, Thailand

**Keywords:** activated carbon, sugarcane bagasse, supercapacitors, nanoparticles, LiPF_6_ electrolyte

## Abstract

Activated carbon (AC) from sugarcane bagasse was prepared using dry chemical activation with KOH. It was then subjected to a high-energy ball milling (HEBM) treatment under various milling speeds (600, 1200 and 1800 rpm) to produce AC nanoparticles from micro-size particles. The AC samples after the HEBM treatment exhibited reduced particle sizes, increased mesopore volume and a rich surface oxygen content, which contribute to higher pseudocapacitance. Notably, different HEBM speeds were used to find a good electrochemical performance. As a result, the AC/BM12 material, subjected to HEBM at 1200 rpm for 30 min, exhibited the highest specific capacitance, 257 F g^−1^, at a current density 0.5 A g^−1^. This is about 2.4 times higher than that of the AC sample. Moreover, the excellence capacitance retention of this sample was 93.5% after a 3000-cycle test at a current density of 5 A g^−1^. Remarkably, a coin cell electrode assembly was fabricated using the AC/BM12 material in a 1 M LiPF_6_ electrolyte. It exhibited a specific capacitance of 110 F g^−1^ with a high energy density of 27.9 W h kg^−1^.

## 1. Introduction

Electric double layer capacitors (EDLCs), or supercapacitors, store energy via double layers consisting of an electrode surface and electrolyte ions. EDLCs have excellent electrochemical performance with high power density, fast charge/discharge and long life-cycles [1,2]. They can store electrostatic charges or ions at their surfaces or in electrode pores with no redox reactions or structural changes, giving them an advantage over batteries and other energy storage devices [3]. Generally, EDLC electrodes are made of porous carbon materials with large surface areas, including carbon nanotubes, graphene, carbon nanofibers and other activated carbon [4,5]. However, a major disadvantage of EDLCs is their low energy density [6]. Additional performance improvements are mainly related to specific energy density. Recently, improvement of EDLCs functionalized by Faradaic reactions for high energy density have become attractive. For example, use of novel electrolytes or some electrochemical reactions on electrode surfaces of supercapacitors has been demonstrated [7,8,9].

Nowadays, carbon-based products from various biomass wastes such as corn stover [10], wood [11], rice straw [12], rice husks [13], and bamboo [14], have attracted extensive interest for energy storage applications [15]. Sugarcane bagasse, one of the most plentiful agricultural biowastes, is a potential alternative source of carbon products. This is due to its great abundance, low cost, biocompatibility and eco-friendly nature [16,17,18,19,20]. In general, two processes are used for preparation of biomass derived activated carbon. They are pyrolysis and chemical activation methods. These are the most common preparation routes to produce refined microporous structures, high pore volumes and large surface areas of activated carbon [21]. Accordingly, activated carbon from sugarcane bagasse is considered a novel material for supercapacitor electrodes [22,23,24]. Nevertheless, high-surface area and micropores in carbon material electrodes are not the only factor needed for high performance [25,26]. Mesopores (pore diameters of 2–50 nm) shorten the transport paths of electrons and ions, which can also enhance the electrical performance of the materials [27].

Recently, nanoscale materials have become a research focus for electric double layer capacitors (EDLCs) in energy storage and conversion applications due to their superior properties compared to materials with micro-macro particle sizes. They have higher surface areas, high pore volumes, faster kinetics, and lower resistance. A top-down method via ball milling is a simple technique used to reduce particle sizes to nanoscale. Additionally, nanoparticles exhibit a larger mesopore volume compared to micrometer sized particles, providing one of the key factors for high performance electrochemical supercapacitors [28]. Furthermore, high-energy ball milling (HEBM) is a novel technique for decreasing particle size as it is a fast grinding process to perform high speed milling with high kinetic energy [29]. Decreased particle sizes enable production of electrodes with increased surface area, resulting in greater levels of ion adsorption on the surfaces and in pores of these materials. This results in supercapacitors with superior capacity due to short ion diffusion pathways and low resistance, among other factors [30,31]. Moreover, mesopores can enhance electrochemical performance [27]. Several research studies demonstrated that high surface area and large mesopores were generated in high-energy ball milling processes [32,33].

Here, we employed an HEBM process to produce AC from sugarcane bagasse by adjusting milling speed to 600, 1200 and 1800 rpm for 30 min. The impact of structure, morphology, porosity, and surface chemical groups of electrode materials on electrochemical performance was examined. Interestingly, we demonstrated that an HEBM process can decrease the particle size of AC samples and enhance surface chemistry. Moreover, the HEBM milling speed was studied to obtain the highest specific capacitance, 257 F g^−1^, at a current density 0.5 A g^−1^. This achieved a capacitance retention of 93.5% after a 3000-cycle test at a current density of 5 A g^−1^ in a sample produced with a milling speed of 1200 rpm. Furthermore, a symmetrical coin cell electrode system was assembled with a 1 M LiPF_6_ electrolyte for a supercapacitor device with high energy density of 27.9 W h kg^−1^.

## 2. Materials and Methods

### 2.1. Synthesis

**Figure 1:**
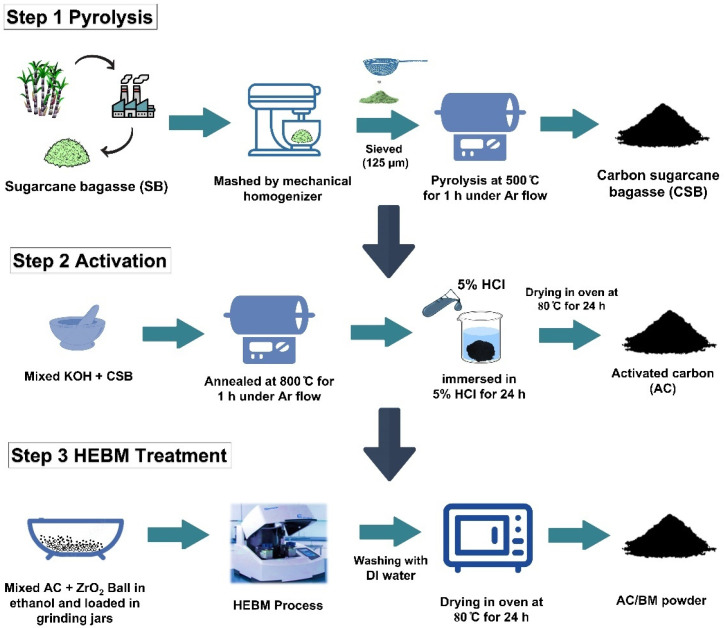
A schematic illustration for the preparation of AC/BM.

Sugarcane bagasse, a biomass waste, was used as a starting raw material. The products were prepared in three steps, carbonization, activation and an HEBM process, as depicted in Figure 1. In the first step, sugarcane bagasse (SB) was shredded and dried at 150 °C for 24 h. Then, the dried SB underwent size reduction using a mechanical homogenizer (JGY-800B, Yongkang hardware capital, Zhejiang, China) at a speed of 25,000 rpm. The resulting material was passed through a 125 µm stainless steel sieve to obtained SB powder. The sieved SB powder was pyrolyzed at 500 °C for 1 h in a quartz-tube furnace at a heating rate of 10 °C min^−1^ under an Ar flow to obtain carbon from sugarcane bagasse (CSB). Pyrolysis temperature was determined from thermal gravimetric analysis (TGA) and derivative thermogravimetry (DTG) (STA7200, HITACHI, Tokyo, Japan) (Figure S1 in the Supporting Information). Then, the CSB powder was mixed with KOH at a weight ratio of 1:4. This condition was optimized to attain the highest electrochemical performance by varying the CSB:KOH ratio as shown in Figures S2 and S3 (Supporting Information). The dried powder was then annealed at 800 °C for 1 h under an argon flow using a heating rate 10 °C min^−1^ to produce activated carbon (AC) powders [21,34]. In the final step, the AC powders were subjected to high-energy ball milling (HEBM) (Model Emax, Retsch, Haan, Germany). 1 g of AC and 20 g of zirconium oxide balls (0.5 mm of diameter) were mixed in 50 mL of ethanol and loaded into a 125 mL grinding jar. The mixture was milled by HEBM at milling speeds of 600, 1200 and 1800 rpm for 30 min, and referenced as the AC/BM6, AC/BM12 and AC/BM18 samples, respectively. After the HEBM process, the products were washed several times with deionized water and subsequently dried at 80 °C in an oven.

### 2.2. Characterization

Thermal gravimetric analysis (TGA) and derivative thermogravimetry (DTG) (STA7200, HITACHI, Tokyo, Japan) were used to determine the pyrolysis temperature of the samples. These measurements were performed starting from room temperature with heating to 900 °C under nitrogen flow (flow rate of 200 mL min^−1^) at a heating rate of 10 °C min^−1^. An X-ray diffraction (XRD) technique (Empyrean, Almelo, The Netherlands) with Cu K_α_ radiation was used to identify crystal structures. Carbon phases were studied using Raman spectroscopy (XploRA plus, Horiba, Kyoto, Japan) with a laser excitation wavelength of 532 nm. The morphology and particle size distributions of samples were examined using field emission scanning electron microscopy (FE–SEM, Helios Nanolab G3 CX, FEI, Brno, Czech Republic) and a Zetasizer nano ZS (Malvern Panalytical, Malvern, MA, USA), respectively. The specific surface area (SSA) and pore size distributions (PSD) were determined using an N_2_ adsorption-desorption method (Micromeritics, 3Flex, Tokyo, Japan) at 77 K. The SSA area was determined using a Brunauer–Emmett–Teller (BET) method. A T-plot was used to calculate the micropore volume (V_micro_). The PSDs of micropores and mesopores were determined using density functional theory (DFT) and a Barrett–Joyner–Halenda (BJH) method, respectively. All samples were degassed for 24 h at 300 °C under a vacuum before measurements were obtained. The sample surfaces were characterized using X-ray photoelectron spectroscopy (XPS) (PHI 5000 versa ProbII@ Ulvac-PHI Inc., Kanagawa, Japan). Fourier transform infrared spectrophotometry (FT–IR) (TENSOR27, Bruker, Osaka, Japan)] was used to identify the functional groups of the materials. The water contact angle on the surface of the aerogels was studied with a specialized instrument (FTA1000 Drop Shape Analysis System, Cambridge, UK).

### 2.3. Electrochemical Measurements

#### 2.3.1. Electrochemical Performance in a Three-Electrode System with 3 M KOH

A three-electrode system with a platinum counter electrode and an Ag/AgCl reference electrode with a 3M KOH electrolyte solution was used to characterize electrochemical properties. For a working electrode, the AC powders (90 wt.%) were mixed with 10 wt.% of polyvinylidene fluoride and dissolved in an *N*-methyl-2-pyrrolidone (NMP) solution. Subsequently, the mixed solution was coated onto a 1.0 × 1.5 cm^2^ Ni foam substrate over a 1 × 1 cm^2^ area before being dried for 24 h in an oven at 80 °C. Finally, the coated Ni foam electrodes were uniaxially compressed at 5 MPa. Cyclic voltammetry (CV), galvanostatic charge/discharge (GCD), and electrochemical impedance spectra (EIS) were measured using a CORRTEST (CS350 Potentiostat/Galvanostat, Corrtest instruments, Wuhan, China). A voltage range of −1.0 to +0.0 V with scan rates ranging from 5–200 mV s^−1^ for the working electrode was used for CV measurements. The GCD experiments were done at current densities ranging from 0.5–30 A g^−1^ to determine the specific capacitance and cyclic stability of the electrodes. The electrical conductivity of the electrodes was measured using an EIS method within a frequency range of 0.1 Hz–100 kHz. For the three-electrode system, the specific capacitance (C_sp_) was calculated from the following equation [35]:

(1) $$C_{\mathrm{sp}}\;\left(\frac{F}{g}\right)=\frac{I\times \Delta t}{m\times \Delta V}$$

where I (Amperes) is the constant current in discharge, m (grams) refers to the weight of the active material within an electrode, ΔV refers to the voltage change during discharge and Δt (seconds) is the time of discharge.

#### 2.3.2. Electrochemical Performance in a Two-Electrode System with a 1 M LiPF_6_ Electrolyte

Electrodes were prepared utilizing Al foil (16 microns thick) as a cathode current collector and copper foil (9 µm thick) as an anode current collector. 0.8 g of the powder sample (AC/BM12), 0.1 g of carbon-black and 0.1 g of PVDF were mixed with 6 mL of NMP to form a slurry. The slurry was then ball-milled for 24 h at 300 rpm. The well-mixed slurry was deposited on the current collector at a thickness of 200 µm using a doctor-blade. After that, the films were pre-heated for 1 h at 90 °C, cut into 5 × 5 cm^2^ squares and compressed at 5 MPa. Then, they were heated again at 120 °C for 1 h to completely dry the pressed films. The dried films were punched into 16 mm diameter disks and stored in a dry box. A 20 mm diameter coin cell system was used with a polypropylene (PP) film as a separator. The coin cells were crimped with an ECCCM-160E-A crimping machine (MTI Corporation, Richmond, CA, USA) at 5 Mpa after adding 100 µL of 1 M LiPF_6_ in an ethylene carbonate–di-methyl carbonate (EC/DMC) organic electrolyte. The specific capacitance of the symmetric coin cells was calculated from the GCD results using the following equation [36]:

(2) $$C_{\mathrm{sp}}\;\left(\frac{F}{g}\right)=\frac{2I\times \Delta t}{m\times \Delta V}$$

where I (Amperes) is the constant current in discharge, m (grams) refers to the weight of the active material within an electrode, ΔV refers to the voltage change during discharge (excluding IR drop) and Δt (seconds) is the time of discharge.

The energy (E) and power densities (P) of both systems were calculated following Equations (3) and (4) [36], respectively.

(3) $$E\left(\frac{\mathrm{Wh}}{\mathrm{kg}}\right)=\frac{C_{\mathrm{sp}}{\left(V_{\max}\right)}^{2}}{8\times 3.6}$$

(4) $$P\left(\frac{W}{\mathrm{kg}}\right)=\frac{E}{\Delta t}$$

where, C_sp_ is capacitance from the GCD results, ΔE is the operating potential window of charge or discharge in Volts, *i* (Amperes) is the applied current, Δt is the discharge time (seconds) and *m* (grams) is the mass of active materials.

## 3. Results and Discussion

### 3.1. XRD and Raman Spectroscopy

**Figure 2:**
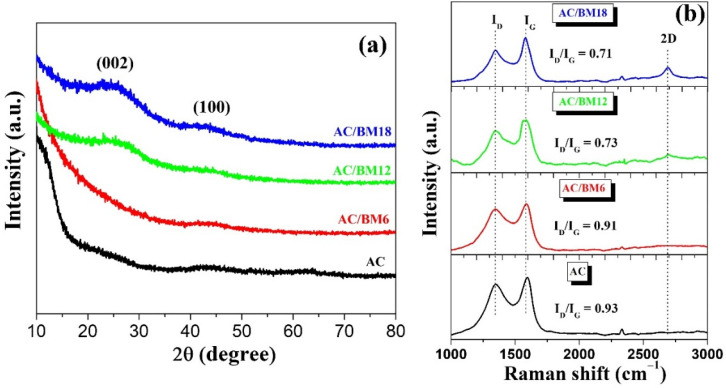
(**a**) The XRD pattern and (**b**) Raman spectra of AC, AC/BM6, AC/BM12 and AC/BM18.

The XRD patterns of AC and AC/BM at various milling speeds of 600, 1200 and 1800 rpm for 30 min are shown in Figure 2a. The AC and AC/BM6 samples reveal no diffraction peak, which indicates an amorphous structure for these samples. However, the AC/BM12 and AC/BM18 samples showed broad 2θ peaks at around 26° and 43°, ascribed to the (002) and (100) planes of a graphite structure [37]. These results suggest that the characteristic graphitic crystal content increased in these samples. Numerous researchers reported that increasing the (002) peak of graphitic carbon can greatly enhance electrical conductivity [25]. The intensity of the broad (002) peaks increased at milling speeds of 1200 and 1800 rpm, suggesting that the HEBM process could induce graphitic structures [38]. The structure of carbon materials was further investigated using Raman spectroscopy [39]. Figure 2b shows Raman spectra of all samples. Two major peaks were observed at Raman shifts of approximately 1340 and 1580 cm^−1^, corresponding to the D-band and G-band, respectively [10,11]. The D-band reveals disorder defects in carbon materials, while the G-band can be attributed to the graphitic structures of carbon materials [12]. The ratios of the relative intensities of the D-band and G-band (I_D_/I_G_) values of AC, AC/BM6, AC/BM12 and AC/BM18 were approximately 0.93, 0.91, 0.73 and 0.71, respectively. The I_D_/I_G_ ratio decreased with increasing HBEM speed, indicating an increased degree of graphitization [40], which is in good agreement with the XRD analysis. Moreover, a sharp narrow 2D peak at about 2700 cm^−1^ was observed for AC/BM12 and AC/BM18, inferring that the samples exhibit some portions of graphene sheets [40,41]. The induced 2D graphite from HBEM could lead to enhanced conductivity [40,41].

### 3.2. FE-SEM and Zeta Particle Size Analysis

**Figure 3:**
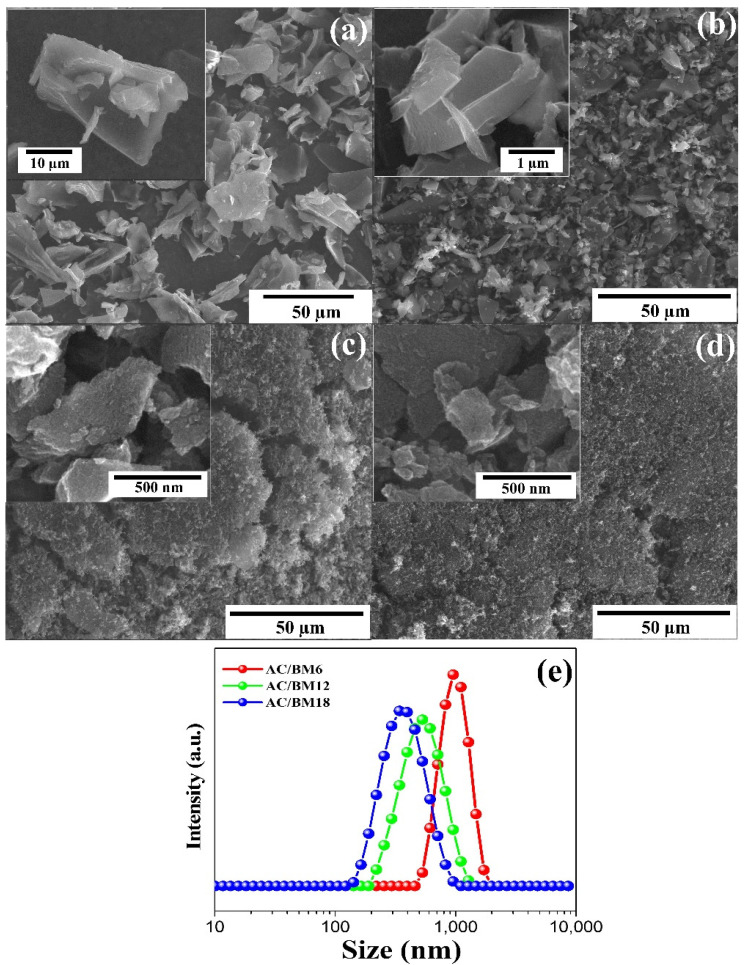
FESEM images of (**a**) AC, (**b**) AC/BM6, (**c**) AC/BM12, (**d**) AC/BM18 and (**e**) Zeta nanoparticle size analysis of AC/BM6, AC/BM12 and AC/BM18.

FE-SEM images in Figure 3a–d show the morphologies of AC and AC/BM. The AC samples revealed large irregularly-shaped particles that are about 10–30 µm in size (Figure 3a). The particle size of AC after HEBM at 600 rpm decreased to 1–5 µm, as shown in Figure 3b. Moreover, after HEBM at speeds of 1200 and 1800 rpm, the samples presented decreased particle diameters to nano-scale sizes, as shown in Figure 3c,d. However, there are some agglomerations in the AC/BM12 and AC/BM18 samples ranging from 100 nm^−1^ µm. A Zeta nanoparticle size analyzer was employed to determine the size distribution of AC/BM6, AC/BM12 and AC/BM18, to quantify the particle size distributions. These results are shown in Figure 3e. The average particle sizes were 1.0 ± 0.11, 0.55 ± 0.13 and 0.39 ± 0.12 µm for AC/BM6, AC/BM12 and AC/BM18 samples, respectively. The results show that increasing the milling speed caused a decrease in the particle size, with average particle size of about 0.4–1 µm, compared to 10–30 µm for the AC powder. The reduction of particle sizes via HEBM is in good agreement with a previous report [26].

### 3.3. N_2_ Adsorption–Desorption

**Figure 4:**
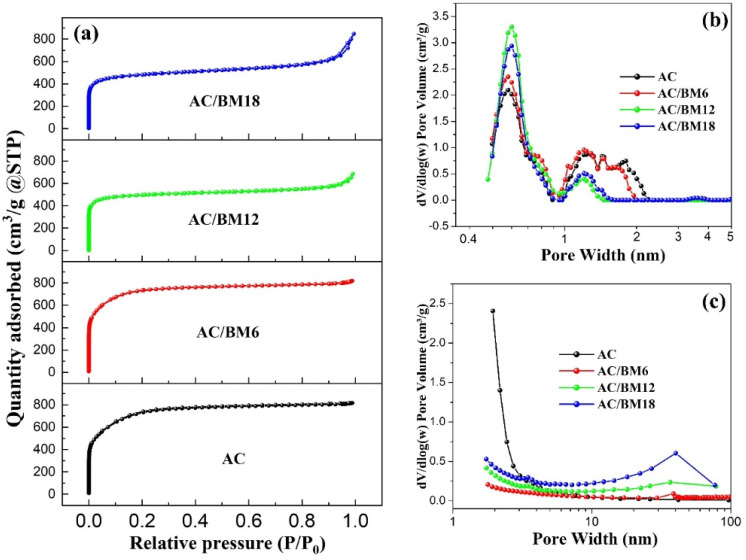
(**a**) N_2_ adsorption–desorption isothermal curves, (**b**) micropore-size distribution and (**c**) mesopore-size distribution of AC, AC/BM6, AC/BM12 and AC/BM18.

To further understand the microstructure of the prepared samples, the specific surface areas and pore size distributions were studied using an N_2_ adsorption–desorption technique. The adsorption-desorption isotherms of all samples are shown in Figure 4a. Isothermal curves of AC and AC/BM6 exhibit typical Type I curves, indicating the presence of large proportions of micro-porous material (<2 nm) in the low-pressure region. This type of isotherm represents materials that exhibit very small porous structures. In contrast, AC/BM12 and AC/BM18 samples display typical Type IV isotherms with H3 type hysteresis loops. This indicates the presence of micropores (<2 nm) and mesopores (2–50 nm) [42]. At a relative pressure of 0.8 in the adsorption branch of AC/BM12 and AC/BM18, the hysteresis loop at P/P_0_ = 0.80–0.99 exhibits a remarkable desorption process, indicating increased mesopore adsorption from carbon wedged-shape pores [42]. The BET surface areas were approximately 2631.9, 2592.4, 1580.9 and 1549.4 m^2^ g^−1^ for AC, AC/BM6, AC/BM12, and AC/BM18, respectively. As a result, the highest specific surface area was obtained in the AC sample because it had the greatest microporous volume. The specific surface area of the AC in this work is higher than in previous reports for activated carbon from sugarcane bagasse [22,37,43]. It is notable that the specific surface areas of the samples after HEBM decreased, which is attributed to a reduction of micropores by the HEBM process. These results are supported by previous research that demonstrated a decreased texture with milling time [44]. Figure 4b shows the pore size distribution of micropores using a DFT method. It was observed that the pore size distribution of all samples is similar, with a major peak ranging from 0.5–0.9 nm and another broad peak at around 1–2 nm. Moreover, the pore volume at a diameter of 0.6 nm for AC/BM12 was higher than in other samples. However, further increasing the milling speed, i.e., to AC/BM18, decreased the micropore volume of the sample due to the impact of HEBM [26]. Mesopore pore size distribution was studied using a BJH method and the results are shown in Figure 4c. At higher milling speeds, the mesopore volume of the HEBM samples (at about 10–50 nm) increased. The specific surface area, pore volume and average pore diameter results are summarized in Table 1. It is notable that the average pore diameter (D_avg_) of 0.48 nm for AC/BM12 is less than those of other samples.

### 3.4. XPS and FT–IR

**Figure 5:**
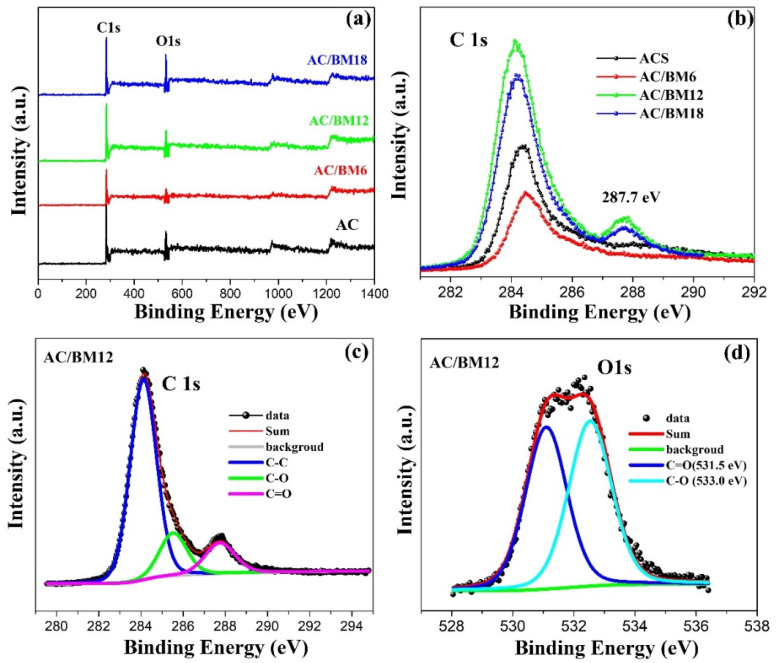
(**a**) Full spectrum of all samples, (**b**) comparison of the C 1s spectra of all samples, (**c**) fitting of C 1s spectrum and (**d**) fitting of O 1s spectrum for AC/BM12.

XPS analysis was employed to further study the effect of the HEBM process on the elemental composition at the AC surface, as shown in Figure 5. A wide scan of the XPS spectra for AC, AC/BM6, AC/BM12 and AC/BM18 is shown in Figure 5a. C 1s (284.60 eV) and O 1s (532.3 eV) peaks are detected in all samples. Narrow XPS scans for the C 1s of all samples are presented in Figure 5b. The intensity of the C-C peak (284.6 eV) is maximal for the AC/BM12 sample. Moreover, increasing the milling speed to 1200 and 1800 rpm resulted in a peak at 287.7 eV, corresponding to the increased presence of C=O groups, indicating enhanced surface oxygen species in these samples [23,45]. These surface oxygen species can increase the wettability (hydrophilicity) of the electrolytes, promoting improved electron and ion adsorption between the surfaces, resulting in better electrochemical performance [9,46,47]. Table 2 summarizes the surface atomic composition and atomic ratios of oxygen and carbon atoms. The oxygen content increases through the HEBM process, from 13.3% to 20.5%, with the O/C ratio increased from 0.186 to 0.258. Moreover, the abundance of surface oxygen is not only a factor promoting hydrophilicity of the AC, but also enhances its electrochemical performance [23,46]. The high-resolution C 1s spectrum of the AC/BM12 sample was fitted with three peaks, as shown in Figure 5c. It consists of graphitic carbon (C-C, 284.6 eV), oxygen of carboxylic groups and/or water (C–O, 285.8 eV) and quinone groups along with carbonyl oxygen (C=O, 287.8 eV). Figure 5d shows the XPS spectrum of O 1s from AC/BM12, which can be fitted to binding energies of 531.5 eV for a C–O bond and at 533.0 eV for a C=O bond. The HEBM treatment induced mesopores in the structure of the AC samples (as shown in Figure 4c and Table 1). The higher mesopore volumes contributed to more oxygen-containing groups, as illustrated in Figure S4 in Supplementary Information. Therefore, the samples with large mesopore volume (AC/BM12 and AC/BM18) also exhibited the large O/C ratio (as shown in Table 2).

**Figure 6:**
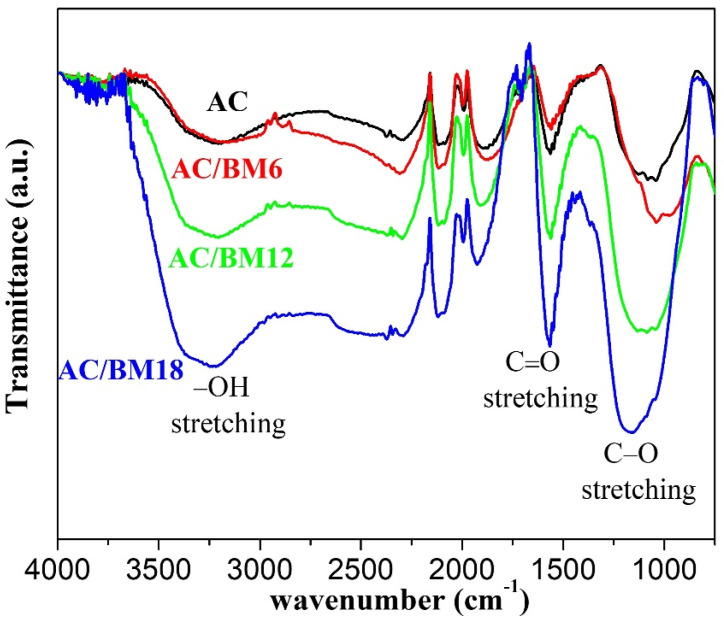
FT-IR spectra of the AC, AC/BM6, AC/BM12 and AC/BM18 samples.

The results from XPS study were confirmed by the FT-IR measurements which show the functional groups of all AC samples, as shown in Figure 6. The oxygen-containing groups are indicated as the -OH stretching at 3100–3500 cm^−1^ [12], the C=O stretching at 1570 cm^−1^, and the C–O stretching at 1150 cm^−1^ [13]. It is clearly seen that the intensity of these absorption bands increases with increasing HBEM speed. This is supporting evidence to the FTIR analysis. However, the quantitative analysis of O/C ratio from the FTIR spectra is not straightforward but requires standard calibration. Therefore, we did not attempt to quantify the O/C ratio. It should be noted that there is a noticeable decrease of the C=O peak for the AC/BM6 sample compared to the AC sample. This could be the origin of the reduced O/C ratio of AC/CM6 in Table 2.

**Figure 7:**
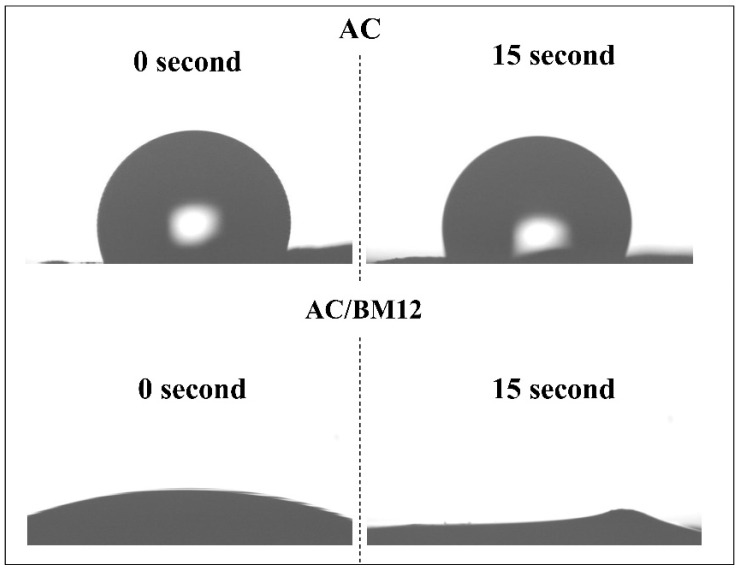
Dynamic water contact angle measurement for AC and AC/BM12 samples at 0 s and 15 s after dropping water.

To confirm the wettability of AC after HEBM process, the dynamic water contact angle measurement was investigated for AC and AC/BM12 samples, as shown in Figure 7. The AC is hydrophobic with the contact angle of 101.9° from the beginning (0 s) to the end (15 s). Meanwhile, the AC/BM12 is hydrophilic. The water drop was absorbed instantly. This result clearly indicates an improved wettability of the AC/BM compared to the AC due to the presence of oxygen-containing groups after the HEBM.

### 3.5. Electrochemical Properties

**Figure 8:**
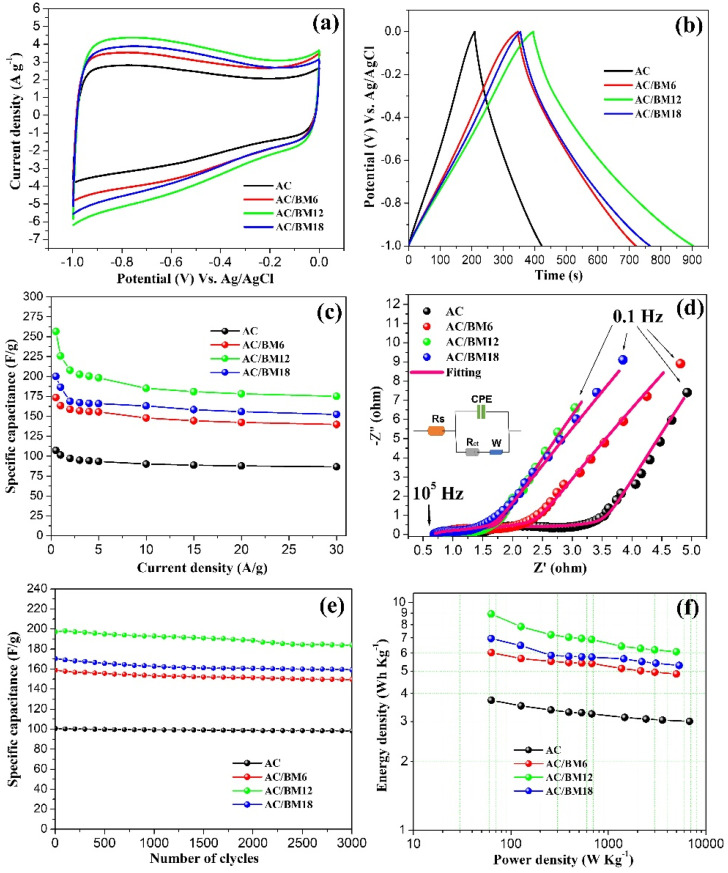
(**a**) CV curves at a scan rate of 20 mV s^−1^, (**b**) GCD curves at a current density of 0.5 A g^−1^, (**c**) specific capacitance (C_sp_) at various current densities, (**d**) Nyquist plots, (**e**) the cycling retention at 5 A g^−1^ and (**f**) Ragone plot of energy density and power density for AC, AC/BM6, AC/BM12 and AC/BM18 electrodes.

To test the effect of KOH activation, the CBS:KOH ratios were varied as 1:1, 1:2, 1:3, 1:4, 1:5, and the electrochemical properties of AC powder from each condition were determined. These results are summarized in the Supporting Information (Figure S3). Optimal electrochemical performance was obtained when a CSB:KOH ratio of 1:4 was used. The highest specific capacitance, 107 F g^−1^, was attained at a current density of 0.5 A g^−1^. Therefore, this condition was selected for further improvement via the HEBM process. After HEBM treatment, the electrochemical properties of AC, AC/BM6, AC/BM12 and AC/BM18 electrodes were examined in a three-electrode system with a 3 M KOH electrolyte, and the results are shown in Figure 8a–f. A comparison of CV curves at a scan rate of 20 mV s^−1^ for AC, AC/BM6, AC/BM12 and AC/BM18 electrodes is given in Figure 8a. It can be clearly seen that the curves show nearly rectangular shapes, indicating that the capacitance mainly resulted from an EDLC behavior [48]. All electrode samples exhibit large storage of charges or ions on their surfaces and in pore sites. Clearly, the CV curve of AC/BM12 electrode contains a larger area than the others, indicating its greater specific capacitance. The highest specific capacitance for the AC/BM12 electrode can be attributed to an appropriate level of micropores and mesopores. Additionally, the average pore diameter of this sample is smaller than others (Table 1), suggesting that smaller pores facilitate better ion transport in EDLC electrodes [49]. Moreover, another reason for the high specific capacitance of AC/BM12 is possibly due to the pseudocapacitance from a Faradaic reaction of oxygen groups and compounds containing C=O and C–O groups [23,45]. The Faradaic pseudocapacitance at the surface by oxygen-containing groups of the electrode in electrolytes can be described by Equations (5) and (6) [9,47].

>C–O ↔ O + H^+^ + e^−^ (Phenolic) (5)

>C=O + e^−^ ↔ >C–O (Ketone) (6)

Galvanostatic charge-discharge measurements of AC, AC/BM6, AC/BM12 and AC/BM18 electrodes were examined at a current density of 0.5 A g^−1^, as shown in Figure 8b. These results indicate triangular-shaped charge/discharge curves for all electrodes, which confirmed electrical double layer formation [24,36]. The highest specific capacitance was found to be 256.6 F g^−1^ for the AC/BM12 electrode. Furthermore, the observed specific capacitance (C_sp_) values of the AC, AC/BM6, AC/BM12 and AC/BM18 electrodes were calculated at various current densities (0.5, 1, 2, 3, 4, 5, 10, 15, 20 and 30 A g^−1^) from the discharge curves using Equation (1), with results presented in Figure 8c. It was found that the C_sp_ values decrease with increased current density. This occurs since the electrolyte ions cannot access the surface or pores quickly enough. Thus, there is a potential drop at higher current densities [31,50]. Interestingly, our results for the electrochemical performance are comparable with previous reports for other biomass-derived carbon sources for supercapacitors, as summarized in Table 3. The outstanding electrochemical performance from the present work demonstrates that AC/BM12 is a promising electrode material for energy storage devices.

The equivalent resistance and charge transfer resistance of all electrodes measured using an EIS technique at the frequency range of 0.1–10^5^ Hz are presented as Nyquist plots in Figure 8d. These results reveal two overlapping semicircular arcs in the high frequency region and straight lines at low frequencies. At low-frequency range, the AC/BM12 appears to have high higher capacitance or lower resistance, which can contribute to the high performance of this electrode. The EIS curves were fitted using an equivalent circuit model, as shown in Figure 8d, where R_S_ is the series resistance that is mainly related to the bulk electrolyte resistance, CPE is the constant phase element related to a charge accumulation at the electrode/electrolyte interface, R_ct_ is the charge-transfer resistance, and W is the diffusion impedance, often called the Warburg Element [48,51]. All fitting parameters are summarized in Table S1 in Supplementary Information. HEBM reduced the particle sizes and induced graphitization, leading to higher conductivity, and thus decreased Rs values of electrodes [40,41]. The AC/BM18 electrode possesses the smallest R_S_ and R_ct_, suggesting that smaller particles and larger mesopores facilitate ion-transport in EDLC electrodes [23,24,50]. However, the small R_S_ and R_ct_ are not the only factor necessary for high specific capacitance. Other parameters play a role in determining the capacitance, such as the effective ohmic resistance of the electrolyte and the charge transfer resistance at the interface, which depend on surface area, pore size distribution and surface chemical composition, among others. Furthermore, the specific capacitance retention at a 5 A g^−1^ current density remains more than 90% in all sample electrodes after 3000 cycles, as shown Figure 8e, indicating good cycling stability for all samples. The energy density (E) and power density (P) of all samples were calculated using Equations (3) and (4), respectively. Figure 8f shows the energy density versus power density curve in Ragone plots. The results show that the AC/BM12 electrode exhibits the highest energy density, 8.91 W h kg^−1^, and the highest power density, 4.98 kW kg^−1^.

**Table 3:** Comparison of the properties of various biomass-derived carbon sources used in supercapacitors.

Material	Specific Surface Area (m^2^ g^−1^)	Specific Capacitance (F g^−1^)	Scan Rate/Current Density	Electrolytes	Cell (3E/2E)	Ref.
Bamboo	1472	146	0.2 A g^−1^	EMIM TFSI	2E	[52]
Corns stalks	540	213	1 A g^−1^	6 M KOH	3E	[53]
Wastepaper	416	180	2 mV s^−1^	6 M KOH	3E	[54]
Banana fibers	1097	74	0.5 A g^−1^	1 M Na_2_SO_4_	3E	[55]
Coconut shells	1874	268	1 A g^−1^	6 M KOH	3E	[56]
Rice husks	2696	112	1 A g^−1^	1 M Na_2_SO_4_	3E	[57]
Corn stalk core	2350	140	1 A g^−1^	3 M KOH	3E	[10]
Corn silk	2285	160	1 A g^−1^	6 M KOH	2E	[58]
Rice straw	396	112	1 A g^−1^	1 M H_2_SO_4_	3E	[57]
Pistachio shells	1009	125	10 mV s^−1^	1 M HNO_3_	3E	[59]
Beer lees	3560	188	1 mA cm^−2^	0.1 M H_2_SO_4_	3E	[60]
Natural wood	2925	200	2 mV s^−1^	6 M KOH	3E	[61]
Onion peels	-	127	0.75 A g^−1^	1 M H_2_SO_4_	2E	[62]
Sugarcane bagasse	684	216	0.2 A g^−1^	2 M KOH	3E	[22]
Sugarcane bagasse	1437	186	0.5 A g^−1^	6 M KOH	2E	[23]
Sugarcane bagasse	1207	228	0.2 A g^−1^	6 M KOH	2E	[24]
Sugarcane bagasse	1150–2632	257	0.5 A g^−1^	3 M KOH	3E	This work
		110	0.25 A g^−1^	1 M LiPF_6_	2E	This work

### 3.6. Coin Cell Devices

**Figure 9:**
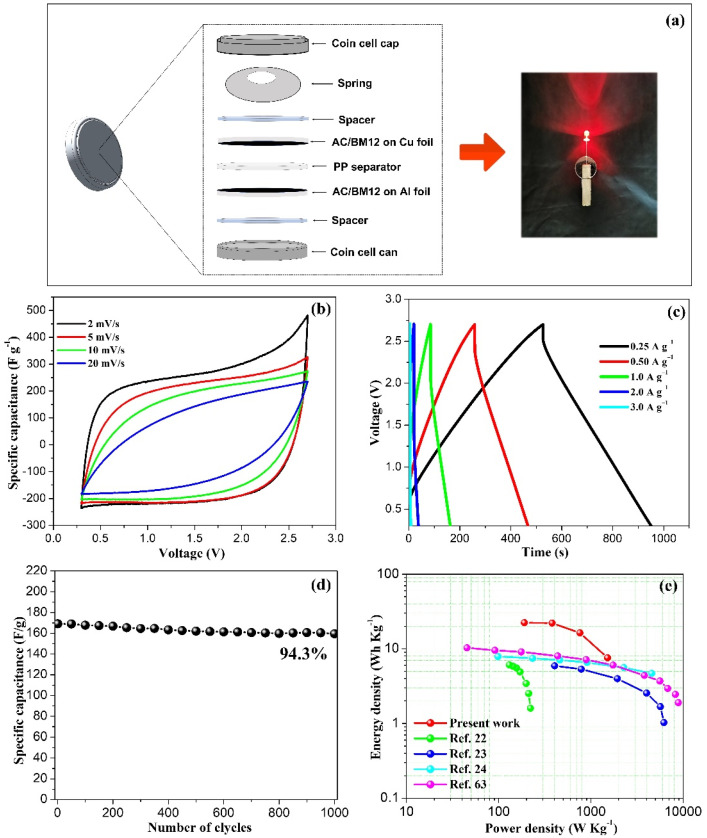
(**a**) a Schematic diagram of a fabricated coin cell powering an LED, (**b**) CV curves, (**c**) GCD curves, (**d**) the cycling retention at 1 A g^−1^ and (**e**) Ragone plot of energy density and power density of AC/BM12 in 1 M LiPF_6_ compared with various porous carbons from sugarcane bagasse in other reported data [22,23,24,63].

To demonstrate a practical application of our AC/BM12 sample for a supercapacitor device, we fabricated coin cells using the synthesized materials as electrodes. Symmetrical electrodes for coin-cells were assembled with a 1 M LiPF_6_ electrolyte to increase the energy density of these devices. This is due to its larger voltage window as the energy density of a supercapacitor is equivalent to the square of the voltage. Figure 9a shows a schematic diagram of a coin cell assembly using an AC/BM12 electrode. It also shows an image of the coin cell connected to an LED, demonstrating its feasibility in a real application. The cyclic voltammograms converted from the current response at CV at scan rates of 2, 5, 10 and 20 mV s^−1^ (Figure 9b) indicate that the CV curves of the sample remained in the form of an ideal rectangular shape, between 0.3 V and 2.7 V. Galvanostatic charge-discharge curves at various current densities of 0.25, 0.50, 1.0, 2.0 and 3.0 A g^−1^ are shown in Figure 9c. The specific capacitance of AC/BM12 in a 1 M LiPF_6_ electrolyte is 110 F g^−1^ at 0.25 A g^−1^ and 27.4 F g^−1^ at 3.0 A g^−1^. Moreover, Figure 9d shows an excellent capacitance retention of 94.3% after 1000 cycles at a 1.0 A g^−1^ current density for this sample. Figure 9e shows Ragone plots of AC/BM12 in a 1 M LiPF_6_ organic electrolyte compared with various porous carbons from sugarcane bagasse. The AC/BM12 electrode in 1 M LiPF_6_ has an energy density of 27.9 W h kg^−1^ at a power density of 204.30 W kg^−1^. It can be seen that the performance of this material is much higher than previously reported. These results show that the AC/BM12 material is a promising electrode material for high performance supercapacitors.

## 4. Conclusions

In summary, we fabricated AC from sugarcane bagasse with improved electrochemical performance using an HEBM treatment. The effect of milling speed on carbon structure, morphology, porosity, surface chemical groups of the AC/BM samples was closely related to the electrochemical properties. It was found that the AC sample ball milled at 1200 rpm (AC/BM12) displayed excellent electrochemical properties with the highest specific capacitance, 257 F g^−1^, at a current density 0.5 A g^−1^. Moreover, the capacitance retention of this sample was 93.5% after a 3000-cycle test at a current density of 5 A g^−1^. The reason for the high specific capacitance of AC/BM12 can be attributed to an increased level of mesopores as well as the presence of a large number of micropores in its structure. Furthermore, the HEBM process induced additional surface functional groups, namely, C=O and C–O, which provided Faradaic pseudocapacitance and abundant adsorption sites for electrolyte ions. These improved the electrochemical performance of the AC/BM12 electrode. Additionally, the AC/BM12 electrodes were assembled in a symmetric coin cell supercapacitor using a LiPF_6_ electrolyte. The device displayed a specific capacitance of 110 F g^−1^ with an energy density of 27.9 W h kg^−1^. An effective HEBM treatment can decrease particle sizes and improve surface chemical groups, which enhanced capacitance. These outstanding electrochemical properties confirm that AC/BM12 is a promising electrode material for supercapacitor applications.

## Figures and Tables

**Table 1:** Porous structural parameters of AC, AC/BM6, AC/BM12 and AC/BM18 samples.

Sample	S_BET_ (m^2^/g)	V_total_ (cm^3^/g)	V_micro_ (cm^3^/g)	V_meso_ (cm^3^/g)	D_avg_ (nm)
AC	2631.9	1.2644	0.6345	0.4814	0.60
AC/BM6	2592.4	1.2680	0.6389	0.3554	0.57
AC/BM12	1580.9	0.9207	0.6318	0.3817	0.48
AC/BM18	1549.7	1.0238	0.5508	0.7017	0.50

**Table 2:** Elemental composition of AC, AC/BM6, AC/BM12 and AC/BM18 samples.

Sample	Atomic Concentration (%)		O/C
	C_1s_	O_1s_	
AC	84.33	15.67	0.186
AC/BM6	86.73	13.27	0.153
AC/BM12	82.22	17.78	0.216
AC/BM18	79.52	20.48	0.258

## Data Availability

The data presented in this study are available on request from the corresponding author.

## Supplementary Materials

The following supporting information can be downloaded at: https://www.mdpi.com/article/10.3390/nano12203555/s1, Figure S1: TGA and DTG curves of sugarcane bagasse powder under argon flow using a heating rate 10 °C min^−1^, Figure S2: The XRD patterns and Raman spectra of CSB and AC samples, Figure S3: (a) CV curves at a scan rate of 20 mV s^−1^, (b) GCD curves at a current density of 0.5 A g^−1^, (c) specific capacitance (C_sp_) at various scan rate and (d) specific capacitance (C_sp_) at various current densities for AC samples, Figure S4: A schematic illustration for the effect of HEBM on the mesopores and the induced oxygen-containing groups of the AC samples., Table S1: Fitting parameters of AC, AC/BM6, AC/BM12 and AC/BM18 electrodes from the Nyquist plots in Figure 8d. Refs. [32,33,34,35] cited in Supplementary Materials.
