# Supplementary material for: Enhanced Electrochemical Performance of Sugarcane Bagasse-Derived Activated Carbon via a High-Energy Ball Milling Treatment
Source: Nanomaterials (Basel). 2022 Oct 11;12(20):3555. doi: 10.3390/nano12203555 (PMC9609200; doi:10.3390/nano12203555)
Supplement: Supplementary file 1 [file nanomaterials-12-03555-s001.zip › nanomaterials-1918589-supplementary.pdf]

Supplementary Information

Enhanced Electrochemical Performance of Sugarcane Bagasse-derived Activated Carbon *via* a High-Energy Ball Milling Treatment

Likkhasit Wannasen, Narong Chanlek, Sumeth Siriroj, Santi Meansiri, Ekaphan Swatsitang, Supree Pinitsoontorn*

Institute of Nanomaterials Research and Innovation for Energy (IN-RIE), Department of Physics, Faculty of Science, Khon Kaen University, Khon Kaen 40002, Thailand;
*Correspondence: psupree@kku.ac.th; Tel.: +66-43-203166; Fax: +66-43-202374

1. **Determination of the pyrolysis temperature for synthesis carbon from sugarcane bagasse (CSB)**

The thermal properties and phase transformation of activated carbon from sugarcane bagasse were studied using TGA and DTG techniques and the results are shown in **Figure S1**. The TGA curve presents three significant weight losses of 4.6%, 15.2% and 55.1% corresponding to the three main peaks of the DTG curve at about 80 °C, 217 °C and 356 °C, respectively. A minor weight loss about 4.6% at temperature below 100 °C is attributed to moisture loss [1]. The weight loss of 15.2 % at temperature from 200–300 °C is ascribed to thermal decomposition of hemicellulose [2]. Then, the major weight loss of 55.1% at temperature from 300–400 °C resulted from cellulose degradation [3]. Moreover, the lignin component in sugarcane bagasse (SB) is significant and appeared between 200 °C to 500 °C overlapping other DTG peaks [4]. Furthermore, no further weight loss or thermal effect was observed at reaction temperatures above 400 °C. Therefore, the SB powder should be pyrolyzed at a temperature higher than 400 °C to form carbon products. That is the reason for the pyrolysis temperature of 500 °C in the present work.

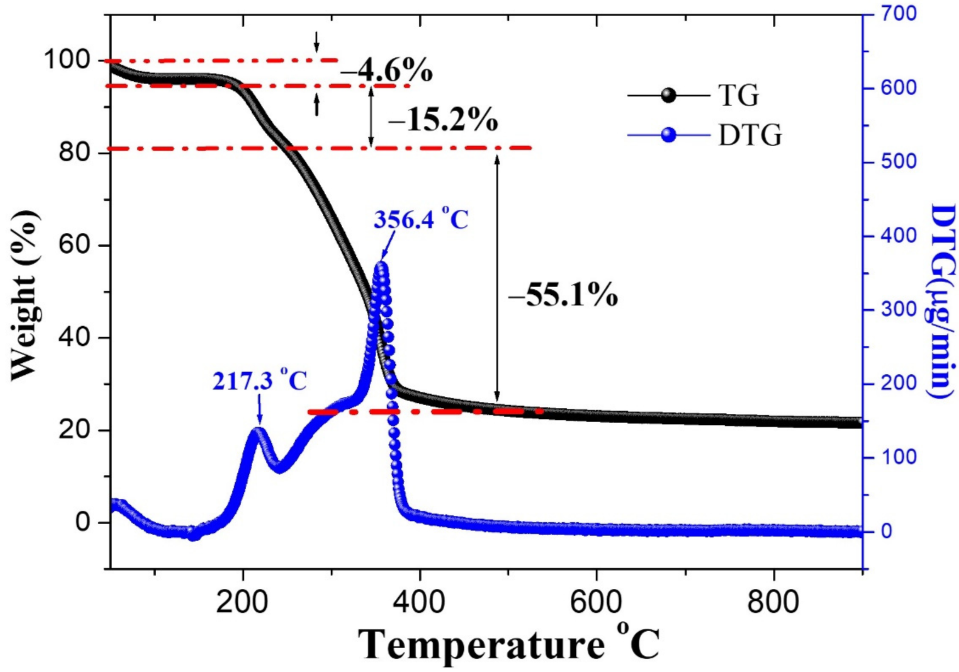

Figure S1 TGA and DTG curves of sugarcane bagasse powder under argon flow using a heating rate 10 °C min^{-1}.

2. Effect of KOH activation

Activated carbon (AC) from sugarcane bagasse was prepared using a dry chemical activation with KOH for supercapacitor electrodes. The synthesis conditions used a mixture of CSB and KOH powder. Then, the dried powder was annealed at 800 °C for 1 h under an argon flow using a heating rate 10 °C min^{-1} for activation. The AC from sugarcane bagasse is referenced as AC1, AC2, AC3, AC4 and AC5 for the optimization study done by varying the CSB:KOH ratios as 1:1, 1:2, 1:3, 1:4 and 1:5, respectively. **Figure S2 (a)** shows the XRD patterns of sugarcane bagasse powder and AC samples. From these results, two dominant peaks at 16.6° and 22.5° reveal a Type I cellulose structure of sugarcane bagasse that can be indexed to the (110) and (200) planes, respectively. Meanwhile, broad diffraction peaks of CSB and all AC samples at 10–80° can be indexed to amorphous carbon. The structures of carbon materials were further investigated using Raman spectroscopy, as shown in **Figure S2 (b)**. The Raman spectroscopy of activated carbon with various KOH ratios exhibit similar adsorption peaks, indicating that all activated carbon samples possess similar carbon structure. However, the intensity of D-band and G-band in AC5 sample is significantly decreased, demonstrating that the carbon structures of activated carbon were destroyed.

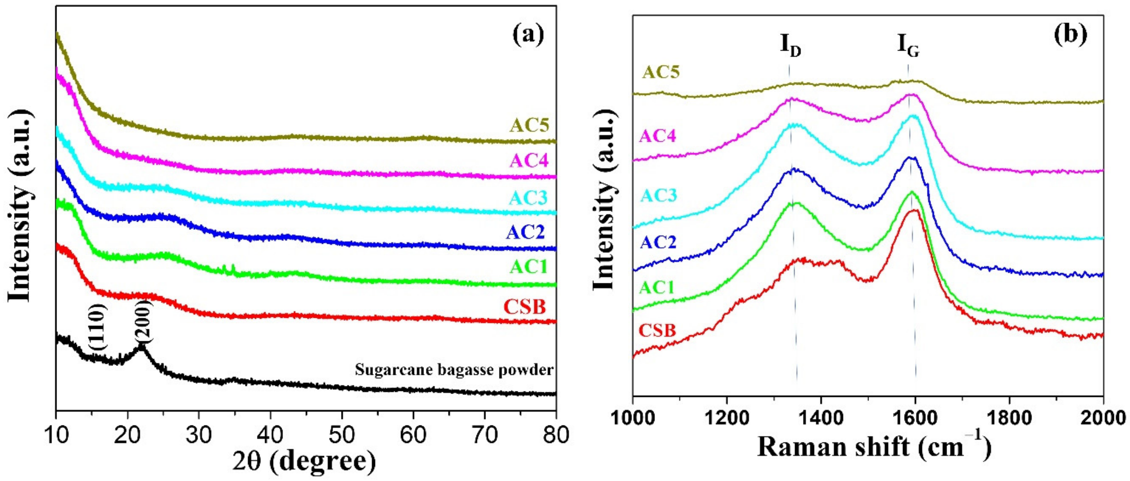

Figure S2 The XRD patterns and Raman spectra of CSB and AC samples.

The electrochemical properties of KOH activation in AC samples were determined in a 3M KOH electrolyte using cyclic voltammetry (CV). **Figure S3(a)** shows comparative CV curves at a scan rate of 20 mV s^{-1} for AC electrodes. From the CV curves, it can be clearly seen that the shapes of the curves show electrical double layer capacitance (EDLC). The Cs values can be calculated from the area under the CV curves and the obtained results shown are in **Figure S3(c).** It was found that the CV curve of AC4 shows the largest area with a higher C_{sp} value (129.4 F/g) than other samples. Galvanostatic charge/discharge (GCD) at a current density of 0.5 A g^{-1} of AC electrodes is shown in **Figure S3 (b)**. Symmetric charge/discharge curves combined with EDLCs were observed. Furthermore, the C_{sp} of each electrode examined using the GCD method was calculated from its discharge curve using **Equation 1**. The results are shown in **Figure S3(d)**. It was found that the AC4 electrode exhibited the highest specific capacitance, 107 F g^{-1}. In summary, the optimal electrochemical performance varied with KOH concentration. The highest performance was obtained in the AC4 sample (CSB:KOH weight ratio of 1:4) by adjusting the ratio of carbon and KOH. The AC4 sample had the highest C_{sp} when it was subjected to a combined high-energy ball milling (HEBM) treatment.

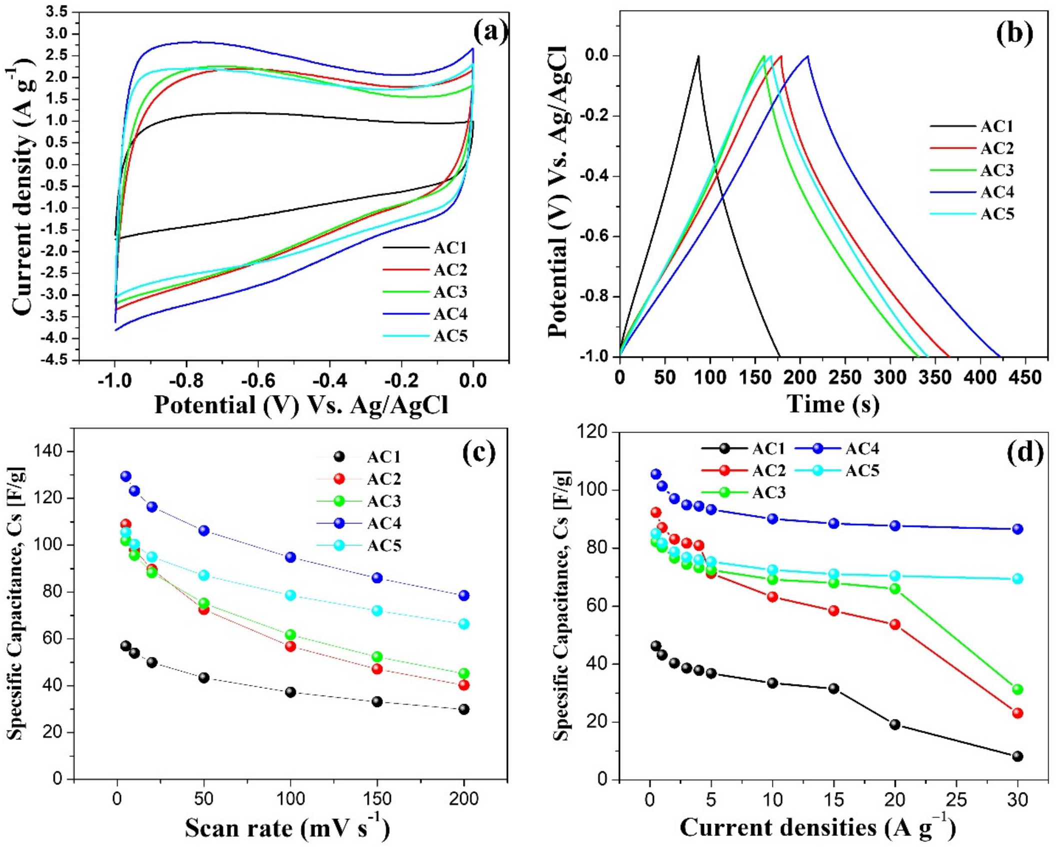

Figure S3 **(a)** CV curves at a scan rate of 20 mV s^{-1}, **(b)** GCD curves at a current density of 0.5 A g^{-1}, **(c)** specific capacitance (C_{sp}) at various scan rate and **(d)** specific capacitance (C_{sp}) at various current densities for AC samples.

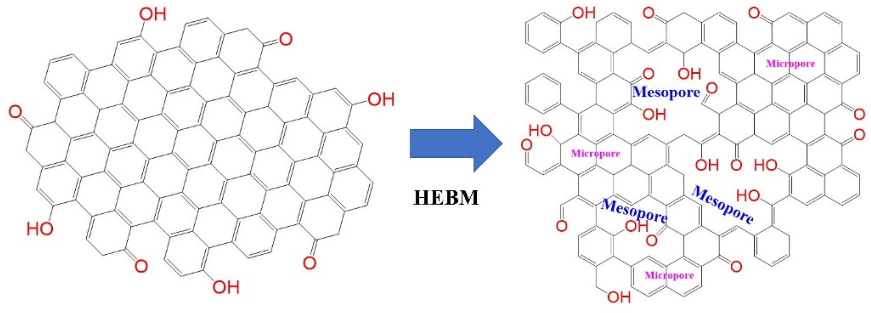

Figure S4 A schematic illustration for the effect of HEBM on the mesopores and the induced oxygen-containing groups of the AC samples.

Table S1. Fitting parameters of AC, AC/BM6, AC/BM12 and AC/BM18 electrodes from the Nyquist plots in **Figure 8(d)**.

Parameters	AC	AC/BM6	AC/BM12	AC/BM18
**Rs**	1.45	0.84	0.81	0.68
**CPE-T (Q)**	0.0106	0.0173	0.0182	0.0164
**CPE-P (n)**	0.5496	0.4542	0.5842	0.6348
**Rct**	1.66	1.08	0.69	0.67
**W-R**	2.1521	1.4725	1.4615	1.6279
**W-T**	0.2655	0.2362	0.2340	0.1905
**W-P**	0.4453	0.4372	0.4462	0.4304

Rs is the series resistance, **CPE-T** is the pseudo-capacitance (**Q**), **CPE-P** is **n** value related to the true capacitance, **Rct** is the charge-transfer resistance, **W-R** is the diffusion impedance, **W-T** is diffusion interpretation and **W-P** is an exponential factor.

References

1. Manikandan, A.; Subramanian, K.; Kannaiyan, P. Effect of high energy ball milling on particle size and surface area of adsorbents for efficient loading of fertilizer. *Asian J. Soil Sci.* **2013**, *8*, 249-254.
2. Hongo, T.; Yoshino, S.; Yamazaki, A.; Yamasaki, A.; Satokawa, S. Mechanochemical treatment of vermiculite in vibration milling and its effect on lead (II) adsorption ability. *Applied Clay Science* **2012**, *70*, 74-78.
3. Meng, L.-Y.; Park, S.-J. Effect of heat treatment on CO2 adsorption of KOH-activated graphite nanofibers. *Journal of Colloid and Interface Science* **2010**, *352*, 498-503.
4. Stoller, M.D.; Ruoff, R.S. Best practice methods for determining an electrode material's performance for ultracapacitors. *Energy & Environmental Science* **2010**, *3*, 1294-1301.
